# Awareness of High Blood Pressure by Nativity Among Black Men: Implications for Interpreting the Immigrant Health Paradox

**DOI:** 10.5888/pcd15.170570

**Published:** 2018-10-04

**Authors:** Helen V. S. Cole, Holly E. Reed, Candace Tannis, Chau Trinh-Shevrin, Joseph E. Ravenell

**Affiliations:** 1 Institut de Ciència i Tecnologia Ambientals, Universitat Autònoma de Barcelona, Barcelona, Spain; 2 Barcelona Lab for Urban Environmental Justice and Sustainability, Institut Hospital del Mar d’Investigacions Mèdiques, Barcelona, Spain; 3 Department of Sociology, Queens College, Queens, New York; 4 Department of Environmental Medicine and Public Health, Icahn School of Medicine at Mount Sinai, New York, New York; 5 Department of Population Health, New York University School of Medicine, New York, NY

## Abstract

**Introduction:**

Differences in the social determinants of health and cardiovascular health outcomes by nativity have implications for understanding the immigrant health paradox among black immigrants. We aimed to understand whether blood pressure awareness, a precursor to achieving blood pressure control among hypertensive patients, varied by nativity among a sample of black men.

**Methods:**

Data were collected from 2010 through 2014. In 2016, we conducted logistic regression models using data from a large sample of urban-dwelling middle-aged and older black men. All men in the study had measured high blood pressure at the time of enrollment and were also asked whether they were aware of having high blood pressure. Independent variables included demographics, socioeconomic status, access to care, and health-related behaviors.

**Results:**

Foreign-born participants were significantly less likely than US-born participants to report awareness of having high blood pressure (*P* < .001). We observed a significant positive relationship between proportion of life spent in the US and being aware of having hypertension (β = 0.863; 95% CI, 0.412–1.314; *P* < .001). This relationship remained after adjusting the model for salient independent variables (β = 0.337; 95% CI, 0.041–0.634; *P = .*03).

**Conclusions:**

Difference in hypertension awareness by nativity may skew surveillance estimates used to track health disparities by large heterogeneous racial categories. Our results also indicate that prior health care experience and circumstances should be considered when studying the immigrant health paradox.

## Introduction

Approximately 1 in 5 hypertensive US adults are unware that they have hypertension (1). Black men have the highest prevalence of premature death and disability from hypertension in the United States (US). The hypertension-related death rate among non-Hispanic black men was 47.1 per 100,000 compared with 17.6 per 100,000 for non-Hispanic white men in 2015 (2), due in part to inequities in blood pressure control (3). Although significant improvement in rates of awareness and treatment of hypertension have been achieved among all groups over the past 2 decades, racial disparities in hypertension control remain (4). In 2010, national estimates indicated that only 49.7% of treated hypertensive black men achieved control compared with 65.0% of treated hypertensive white men (4).

These data come from surveillance efforts that assume homogeneity of racial groups despite the increasing diversity in nativity and ethnicity among black communities, especially in urban areas of the US (5). In addition, the social, economic, and other political determinants of health, which are the underlying causes of health inequities by race, also vary substantially by nativity. Past research on health disparities by nativity has primarily focused on Latino and Asian groups, the largest foreign-born minority groups in the US. However, a growing body of research suggests that trends of better health among foreign-born individuals, known as the “immigrant health paradox,” may also hold for foreign-born blacks (6–8).

Inequities in health outcomes by nativity, including hypertension and awareness of hypertension, may be influenced by differences between US-born and foreign-born blacks in the social and behavioral determinants of health, such as socioeconomic status, health-related behaviors, access to care, and the effects of racism or other types of discrimination (6,9,10). Immigration status and acculturation may also affect the health of black immigrants and, in turn, may characterize inequities in outcomes by nativity (6,9–13). We used data from a large sample of urban-dwelling, middle-aged and older black men who participated in a community-based research study to evaluate the role of nativity and other social determinants in hypertension awareness. We also aimed to understand the implications of our findings for public health surveillance methods and health disparities research.

## Methods

We conducted secondary analyses of data from a large community-based research program that focused on health disparities affecting older black men. The Men’s Health Initiative (MHI), described elsewhere, consists of 2 community-based randomized control trials. MISTER B (14,15) and FAITH-CRC, both tested 2 behavioral interventions for older black men: a patient navigation intervention to encourage colorectal cancer screening and a motivational interviewing intervention to improve blood pressure control. Eligibility criteria for these 2 studies were 1) self-identifying as black or of African descent, 2) being male, 3) being aged 50 years or older, 4) not having a timely screening for colorectal cancer, 5) having high blood pressure at the time of screening, 6) having English language ability, and 7) having a working telephone. Having high blood pressure was defined by the JNC-7 criteria for non–clinic-based measurements as systolic blood pressure of 135 mm Hg or higher or diastolic blood pressure of 85 mm Hg or higher, with lower thresholds of 130 and 80 mm Hg, respectively, for those with comorbid diabetes or kidney disease (16). The phrase “black or of African descent” was used, because the initial use of “black or African American” left some individuals who identified as Afro-Caribbean or African uncertain of their inclusion; they believed “black” meant “African American,” as it is commonly used in the US. Older men (aged ≥50 y) were selected because of their risk for high blood pressure and other chronic diseases, thus the importance in understanding the social context of risk for this population. Both studies were approved by the New York University School of Medicine institutional review board.

Data for the MHI were collected from 2010 through 2014 via recruitment from churches, barbershops, mosques, social service organizations, senior centers, and community health fairs in all 5 boroughs of New York City; recruitment methods are described elsewhere (15). Analysis for this study was conducted in 2016. Eligibility was determined using a short interviewer-administered survey and the average of 3 consecutive blood pressure readings. A total of 1,191 participants enrolled in the 2 studies. Of participants enrolled in the MHI, 9 had enrolled more than once, the second record for each of the 9 participants was omitted. Participants were also excluded from analyses if they were missing data on nativity (n = 14) or ethnicity/region of origin data (n = 7), had “other” regions of origin (n = 12), or were missing data for the primary outcome (n = 5). Multiple imputation using the MI CHAINED command in STATA (StataCorp LP) was performed to generate 20 imputed data sets with complete data for all covariates. To reach convergence on all imputation regression models, participants were also excluded if they were missing data on employment (n = 5), having a personal doctor (n = 1), or language spoken at home (n = 2). The final imputed data set included 1,136 participants.

### Outcome and independent variables

Because all participants had measured high blood pressure at the time of enrollment, the outcome evaluated in this analysis was awareness of high blood pressure, which was determined by the question, “Has a doctor or health care provider ever told you that you have high blood pressure?” Key independent variables were nativity (born in the US or outside of the US) and proportion of life lived in the US (foreign-born only; for participants born in the US the proportion was 1). Demographic control variables were age, marital status, language spoken at home, and religion. Socioeconomic status control variables were highest level of education, employment status, and poverty group based on annual household income. We created a categorical income measure based on the 2011 US Department of Housing and Urban Development’s income limit requirements for housing assistance (determined by area median income, taking into account variation in livable income requirements by geographic area, which is not accounted for in national poverty level) (17).

Access to care was measured using 3 variables: having a personal doctor or primary care provider, having insurance, and perceived discrimination in health care. Perceived discrimination was approximated using the suspicion subscale of the Group-Based Medical Mistrust Scale (GBMMS) (18), which measures an individual’s perceptions of the treatment provided to individuals of the respondent’s own ethnic or racial group, suspicion of mainstream health care systems, and suspicion of health care professionals. This measure had high internal consistency (α = 0.87 for the full GBMMS and α = 0.89 for the suspicion subscale) and evidence for strong content validity supported among a similar population (19). Health-related behaviors were smoking, heavy alcohol use (defined as having more than 2 drinks per day), diet (average fruit and vegetable intake), physical activity (low, [not meeting criteria for either moderate or vigorous activity], moderate [at least 20 min/d of vigorous activity on 3 or more d/wk or at least 30 min/d of moderate activity on at least 5 d/wk], or high [vigorous activity on 3 or more d/wk], measured using the IPAQ-S (International Physical Activity Questionnaire, short version) and health care use behavior.

### Descriptive statistics

All analyses were conducted with STATA (StataCorp LP) version 14. We used descriptive statistics conducted on the first imputed data set using the MI XEQ command to examine the distribution of each independent variable by nativity. Chi-squared tests were used to determine whether there were significant differences in the distributions by nativity for categorical and dichotomous outcomes. For continuous outcomes, analysis of variance tests were used to determine whether there were significant differences by nativity.

### Regression analysis

To take into account the various levels of influence of the categories of variables used in our analysis, hierarchical multiple logistic regression was used to adjust for independent variables. The MI ESTIMATE command was used to include the averages of the 20 imputed data sets for missing values. The first model included only nativity and demographic variables as predictors, and subsequent models included socioeconomic status, access to care, and health-related behavior variables.

## Results

Foreign-born participants were less likely than US-born participants to be aware of having high blood pressure (57.8% vs 70.0%, *P* < .001). Foreign-born participants were generally older than US-born participants (*P* < .001), were more likely to be married or living with a partner (46.4% vs 18.7%, *P* < .001), had a higher level of education (17.2% vs 10.1% with a college degree), were more likely to be employed (36.4% vs 24.7%), were less likely to have insurance (57.5% vs 85.0%, *P* < .001), and were more likely to have never smoked (38.0% vs 15.4%, *P* < .001) (Table 1). Among the sample, 40.6% had no personal doctor and 22.4% had no insurance. Foreign-born participants had been in the US on average for 25 years (standard deviation [SD] = 13.0), or on average approximately 42% (SD = 22%) of one’s life. 

**Table 1:** Characteristics of Study Sample (N = 1,136), by Nativity, Imputed Totals, Men’s Health Initiative, New York City, 2010–2014

Characteristic	Nativity			*P* Value^a^
	Total (N = 1,136)	US-born (n = 829)	Foreign-born (n = 311)	
**Demographics**				
**Mean age, y (SD)^b^**	57.6 (6.6)	56.8 (6.2)	59.8 (7.3)	<.001
**No. of years in the United States**	NA	NA	25.0 (13.0)	NA
**Proportion (SD) of life lived in the United States**	0.84 (0.28)	1.00	0.42 (0.22)	NA
**Language spoken at home**, %				
English	91.5	97.8	74.4	<.001
Some other language	8.5	2.2	25.6	
**Religion**, %				
Christianity	74.7	73.5	77.9	<.001
Islam	13.8	15.9	8.1	
Atheist/agnostic	8.8	9.1	8.1	
Other	2.7	1.6	5.8	
**Marital status, %**				
Married or partnered	26.2	18.7	46.4	<.001
Divorced or separated	29.3	29.6	28.6	
Widowed	7.6	8.1	6.2	
Never married	36.9	43.7	18.8	
**Socioeconomic Status**				
**Education, %**				
Less than high school	31.6	30.6	34.1	.002
High school graduate	38.0	39.8	33.1	
Some college	18.4	19.4	15.6	
College or higher	12.1	10.1	17.2	
**Employment status, %**				
Employed	27.9	24.7	36.4	<.001
Unemployed	44.2	45.0	41.9	
Retired	13.9	13.4	15.3	
Unable to work	13.8	16.5	6.5	
Other	0.3	0.4	0.0	
**HUD poverty level^c^, %**				
Extremely low (≤30%)	76.0	76.7	74.0	.13
Very low (31%–50%)	13.8	12.9	16.2	
Low (51%–80%)	5.6	5.2	6.8	
>80%	4.6	5.2	2.9	
**Access to Care**				
**Has a personal doctor, %**				
Yes, one or more	59.5	61.2	54.9	.05
No	40.6	38.8	45.1	
**Has insurance, %**				
Yes	77.6	85.0	57.5	<.001
No	22.4	15.0	42.5	
**GBMMS Suspicion Subscore (SD)**	2.25 (0.83)	2.23 (0.82)	2.32 (0.84)	.10
**Health-Related Behaviors**				
**Servings per day of fruits and vegetables, %**				
None	3.7	3.7	3.6	.84
1–4	67.1	67.6	65.9	
≥5	29.2	28.7	30.5	
**Physical activity, IPAQ (short) category, %^d^**				
Low	23.8	22.6	27.3	.05
Moderate	24.9	24.0	27.3	
High	51.3	53.4	45.4	
**Didn’t get health care when needed, %**	29.3	29.8	27.9	.54
**Smoking status, %**				
Never	21.6	15.4	38.0	<.001
Current smoker	54.0	63.1	29.6	
Former smoker	24.5	21.5	32.5	
**Has more than 2 alcoholic drinks/d, %**	5.5	6.2	3.6	.09
**Aware of high blood pressure, %**	66.7	70.0	57.8	<.001

Abbreviations: GBMMS, Group-Based Medical Mistrust Scale; HUD, US Department of Housing and Urban Development; IPAQ-S, International Physical Activity Questionnaire, short form; NA, not applicable; SD, standard deviation.

a *P* values calculated using χ^2^ tests, except where indicated.

b Age averages and SDs are reported as unweighted. *P* value calculated using simple linear regression.

c Determined by % of median income.

d Low was defined as not meeting criteria for either moderate or vigorous activity; moderate as participating in at least 20 min/d of vigorous activity on 3 or more d/wk or at least 30 min/d of moderate activity on at least 5 d/wk; and high as participating in vigorous activity on 3 or more d/wk.

In regression Model 1, adjusting for demographic variables only, we observed a significant positive relationship between proportion of life spent in the US and being aware of having hypertension (β = 0.863; 95% CI, 0.412–1.314; *P* < .001) (Table 2). This relationship remained after adjusting for socioeconomic status in Model 2, access to care in Model 3, and health-related behaviors in Model 4. In the fully adjusted model (Table 3), variables with significant relationships with hypertension awareness in addition to proportion of life in the US included being unable to work (β = 0.619; 95% CI, 0.121–1.117; *P* = .01), not having a personal doctor (β = −0.713; 95% CI −0.998 to −0.428; *P* < .001), and not seeking medical care when needed (β = 0.337; 95% CI, 0.041–0.634; *P* = .03).

**Table 2:** Relationship Between Proportion of Life Spent in the United States and Awareness of High Blood Pressure (N = 1,136), Hierarchical Logistic Regression Model, Men’s Health Initiative, New York City, 2010–2014

Model No.	Hypertension Awareness	
	β (95% Confidence Interval)	*P* Value
1^a^	0.863 (0.412–1.314)	<.001
2^b^	0.748 (0.282–1.215)	.002
3^c^	0.744 (0.245–1.243)	.003
4^d^	0.801 (0.282–1.319)	.002

a Adjusted for demographics (age, marital status, language spoken at home, and religion).

b Adjusted for demographics and socioeconomic status (education, poverty level, employment status).

c Adjusted for demographics, socioeconomic status, and access to care (insurance, having a personal doctor, and suspicion subscale of the group-based medical mistrust scale).

d Adjusted for demographics, socioeconomic status, access to care, and health-related behaviors (physical activity, fruit and vegetable intake, smoking, heavy drinking, and not seeking care when needed).

**Table 3:** Relationship Between Proportion of Life Spent in the United States and Awareness of High Blood Pressure (N = 1,136), Complete Hierarchical Regression Model (Model 4) Men’s Health Initiative, New York City, 2010–2014

Covariate	β for Model 4 (95% CI)	*P* Value
**Demographics**		
Proportion of life spent in the United States	0.801 (0.282 to 1.319)	.002
**Age, y**	–0.001 (−0.025 to 0.024)	.96
**Marital status**		
Married	1 [Reference]	
Divorced	–0.341 (−0.704 to 0.022)	.06
Widowed	–0.207 (−0.767 to 0.353)	.47
Never married	–0.136 (−0.499 to 0.226)	.46
**Language spoken at home**		
English	1 [Reference]	
Other	0.287 (−0.206 to 0.779)	.25
**Religion**		
Christianity	1 [Reference]	
Islam	0.024 (−0.370 to 0.418)	.90
Atheist/agnostic	–0.247 (−0.710 to 0.216)	.30
Other	–0.056 (−0.850 to 0.738)	.89
**Socioeconomic Status**		
**Education**		
College graduate or more	1 [Reference]	
Some college	0.023 (−0.462 to 0.509)	.92
High school graduate	0.102 (−0.351 to 0.556)	.66
Less than high school graduate	–0.030 (−0.505 to 0.445)	.90
**HUD poverty level**		
Extremely Low (≤30%)	0.442 (−0.316 to 1.199)	.25
Very Low (31%–50%)	0.451 (−0.340 to 1.241)	.26
Low (51%–80%)	0.041 (−0.814 to 0.896)	.92
>80%	1 [Reference]	
**Employment**		
Employed	1 [Reference]	
Unemployed	0.294 (−0.047 to 0.636)	.09
Retired	0.408 (−0.090 to 0.906)	.11
Unable to work	0.619 (0.121 to 1.117)	.01
Other	0.978 (−1.508 to 3.464)	.44
**Access to Care**		
**Uninsured**	–0.042 (−0.386 to 0.303)	.81
**Has a personal doctor**		
Yes, one or more	1 [Reference]	
No	–0.713 (−0.998 to −0.428)	<.001
**GBMMS score**	–0.152 (−0.317 to 0.014)	.07
**Health-Related Behaviors**		
**Physical activity^a^**		
High	1 [Reference]	
Moderate	0.271 (−0.060 to 0.602)	.11
Low	0.138 (−0.197 to 0.474)	.42
**Fruit and vegetable intake**		
≥5	1 [Reference]	
1–4	0.255 (−0.038 to 0.548)	.09
None	0.523 (−0.274 to 1.321)	.20
**Smoking status**		
Never	1 [Reference]	
Former	0.106 (–0.287 to 0.498)	.60
Current	–0.024 (–0.379 to 0.331)	.89
**Drinking**		
Drinks less than 2 drinks/d	1 [Reference]	
Drinks heavily	–0.016 (–0.587 to 0.556)	.96
**Health care use**		
Did get care when needed	1 [Reference]	
Didn’t get care when needed	0.337 (0.041 to 0.634)	.03

Abbreviations: CI, confidence interval; GBMMS, Group-Based Medical Mistrust Scale; HUD, US Department of Housing and Urban Development.

a Categories determined using the International Physical Activity Questionnaire, short form: low, not meeting criteria for either moderate or vigorous activity; moderate, participating in at least 20 min/d of vigorous activity on 3 or more d/wk or at least 30 min/d of moderate activity on at least 5 d/wk; high, participating in vigorous activity on 3 or more d/wk.

## Discussion

 We found that, among MHI participants, foreign-born participants were less likely to be aware of having hypertension than native-born participants, and proportion of life spent in the US was significantly positively associated with being aware of having hypertension (*P = .*002). Hypertension can effectively be treated and controlled with medication and through lifestyle changes. The low awareness among foreign-born participants may lead to an increase in burden of hypertension-related outcomes that result from uncontrolled high blood pressure. The results of our study may shed light on our previous finding that, among 1,035 black participants, foreign-born black participants had significantly higher blood pressure than those who were born in the US (20). Our results have implications for both public health surveillance efforts and research intended to determine whether the immigrant health paradox holds among black immigrants.

Although inaccuracies in self-reported measures, particularly those for asymptomatic conditions such as hypertension, are important for surveillance efforts among all populations, our study indicates that such errors are not random but are correlated with nativity and years in the US, and thus may cause inaccuracies in tracking and measuring health outcomes and disparities. This trend may cause errors in surveillance efforts, causing low estimated prevalence of hypertension among foreign-born black individuals and among the black race in general, which is noted as having higher mortality rates and worse outcomes related to uncontrolled high blood pressure (4). To fully understand trends in hypertension rates in the general population in surveillance reporting, these nuances must be considered.

In line with studies of other immigrant groups on the immigrant health paradox, several studies document distinct health advantages among foreign-born black individuals. For example, black people who are foreign-born have lower body mass indexes (21) and lower rates of diabetes (22), self-reported high blood pressure (11), and allostatic load (13) than their US-born counterparts. Particularly with regard to hypertension, a largely asymptomatic condition, these findings may be affected by awareness of one’s condition, which is in turn a reflection on prior health care use and experience and other social determinants of health known to vary by nativity.

However, our results may indicate limitations in studies on self-reported blood pressure that document support for the immigrant health paradox, as these studies assume that error in the outcome measure would not vary substantially by nativity. For example, one study found that self-reported hypertension increased with length of time in the US and that foreign-born black individuals who had been living in the US for 10 or more years had 58% higher odds of reporting hypertension than foreign-born non-Hispanic white individuals (23). These results may instead reflect a change in awareness of having hypertension rather than an actual change in prevalence.

We also examined the contributions of several social determinants of health to inequities by nativity in awareness. Research has found that among low income black individuals in the US, foreign-born men are much less likely than their US-born counterparts to report being current smokers (24). In studies examining experiences of racism by nativity among black individuals in the US, foreign-born black individuals are less likely to report experiencing racism (25,26). Furthermore, in one study, foreign-born pregnant black women were more likely to report experiences of racism if they had immigrated before the age of 18 (25). Because racism is a fundamental cause of health disparities (27), these variations in the experiences of racism by nativity and time in the US may have implications for health outcomes. However, we found that, although many of these determinants did vary by nativity, differences in hypertension awareness remained after adjusting for these measures, indicating that additional unmeasured contextual factors may lead to the observed differences.

Current access to health care and preventive services as well as trajectory of health care access and utilization both affect one’s health status, particularly when moving between countries and health care systems. Black immigrants in the US come from different places and regions in the world and under varying circumstances. As more research investigates the epidemiological transition occurring in many parts of Africa and other developing regions from which black immigrants arrive, understanding of risk for chronic disease by place of birth changes. However, in many countries in which health care systems lack sufficient trained medical professionals, population-wide services for chronic disease screening are not possible, which may lead to the potential inequity in awareness of chronic disease by place of origin. Once in the US, lack of access to care because of logistic, economic, or cultural exclusion may further restrict new immigrants from receiving preventive care and screening for chronic diseases. Fear of deportation and other forms of discrimination, which may be increasing given recent trends, may be a serious barrier to obtaining care among immigrants.

Immigration status, visa type, and acculturation may also affect the health of black immigrants. For example, legal, nondisabled, nonpregnant adult immigrants are barred from accessing Medicaid for 5 years after initial entry to the US (28). Some states appropriate state-based funds to cover these individuals, and those with refugee status have access to Medicaid and Medicare. Immigrants are also less likely than US-born individuals to have access to employer-sponsored health insurance (29). After the passage of the Affordable Care Act (ACA) in 2010, permanent residents meeting income requirements were able to access subsidized insurance through the health exchanges regardless of time in the US (30). However, undocumented immigrants remain unable to access health insurance coverage through the ACA, which may contribute to continued disparities in chronic disease diagnosis and management. Among our sample, foreign-born black men had less access to care despite having been in the US for 25 years on average. Current political actions in the US bring even more structural and emotional barriers to accessing care, particularly because of the risk of deportation among undocumented foreign-born individuals. The fear of deportation in addition to variation in insurance status by immigration characteristic further polarizes an individual’s experiences with health care in the US.

Our study had several limitations. Our sample included only men with uncontrolled blood pressure at the time of study recruitment, so we were unable to compare rates of awareness to studies that include men who are successfully treated. Also, participants had blood pressure taken only at one point time, so they did not necessarily have diagnosable hypertension, for which a formal diagnosis requires multiple assessments by a physician. Unaware participants with high blood pressure readings were given additional information and were referred to physicians if they did not have a personal doctor. Among our sample, 40.6% had no personal doctor and 22.4% had no insurance, indicating low levels of access to care, which may have led to low levels of awareness among this sample as a whole.

Our study also relied on data collected for 2 studies for which recruitment was not random. Therefore, our results may have decreased internal validity that resulted from the use of a convenience sample in which participants may have been more willing than people in the general population to participate in the parent trials. However, our use of true community-based recruitment, in which participants came from all 5 boroughs of New York City from various recruitment venues, aimed to ensure a diverse sample. In fact, our study included many participants representative of those who may not have been included in other surveillance efforts that use random digit dialing or other methods for sample selection of hard-to-reach populations (31). Our sample was large and narrowed to only men aged 50 years or older, who are at high risk for high blood pressure and other conditions and who may be targeted by screening and prevention efforts.

Finally, our sample included a small number of recent immigrants, so we did not robustly measure acculturation. Studies of the effect of acculturation on health among black immigrants show mixed results. Dey and Lucas found that selected risk factors and chronic diseases did not differ by length of stay in the US for foreign-born black individuals (32). However, other studies report that the trend among immigrant groups of health status declining with increased time in the US may hold for foreign-born black individuals (23,33). Also, we were unable to determine whether recent immigrants were not recruited due to not presenting with blood pressure readings or because they were less likely to be encountered or to agree to be screened or recruited for the studies. In either case, to prevent disparities between foreign-born and native-born populations, efforts to include recent immigrants in research and in screening and prevention services should be considered. Taken together, these limitations may threaten the generalizability of our results.

Our findings that foreign-born black men were overall less likely than US-born black men to be aware of having high blood pressure have substantial implications for public health surveillance efforts and for research on health inequities among immigrants. In traditional US health disparities literature, black individuals are considered as a homogenous racial group despite diversity by nativity, ethnicity, and experiences of the social determinants of health. Therefore, within-race differences by nativity among older black men may skew results of studies that use traditional racial categories or mask additional disparities in a population that may be less likely to reap the benefits of preventive measures.
